# TRoponin of Unknown origin in STroke evaluated by multi-component cardiac Magnetic resonance Imaging – The TRUST-MI study

**DOI:** 10.3389/fcvm.2022.989376

**Published:** 2022-09-30

**Authors:** Annerose Mengel, Lilyana Nenova, Karin A. L. Müller, Sven Poli, Markus C. Kowarik, Katharina Feil, Lars Mizera, Tobias Geisler, Jens Kübler, Heiko Mahrholdt, Ulrike Ernemann, Florian Hennersdorf, Ulf Ziemann, Konstantin Nikolaou, Meinrad Gawaz, Patrick Krumm, Simon Greulich

**Affiliations:** ^1^Department of Neurology and Stroke, Hertie Institute for Clinical Brain Research, University of Tübingen, Tübingen, Germany; ^2^Department of Cardiology and Angiology, University of Tübingen, Tübingen, Germany; ^3^Department of Neurology, Technische Universität München, Munich, Germany; ^4^Department of Diagnostic and Interventional Radiology, University of Tübingen, Tübingen, Germany; ^5^Department of Cardiology and Angiology, Robert Bosch Medical Center, Stuttgart, Germany; ^6^Department of Diagnostic and Interventional Neuroradiology, University of Tübingen, Tübingen, Germany

**Keywords:** ischemic stroke (IS), troponin, CMR (cardiovascular magnetic resonance), inflammation, CAD, myocardial scar

## Abstract

**Aims:**

Increased high-sensitive cardiac troponin I (hs-cTnI) levels are common in patients with acute ischemic stroke. However, only a minority demonstrates culprit lesions on coronary angiography, suggesting other mechanisms, e.g., inflammation, as underlying cause of myocardial damage. Late Gadolinium Enhancement (LGE)-cardiac magnetic resonance (CMR) with mapping techniques [T1, T2, extracellular volume (ECV)] allow the detection of both focal and diffuse myocardial abnormalities. We investigated the prevalence of culprit lesions by coronary angiography and myocardial tissue abnormalities by a comprehensive CMR protocol in troponin-positive stroke patients.

**Methods and results:**

Patients with troponin-positive acute ischemic stroke and no history of coronary artery disease were prospectively enrolled. Coronary angiography and CMR (LGE, T1 + T2 mapping, ECV) were performed within the first days of the acute stroke. Twenty-five troponin-positive patients (mean age 62 years, 44% females) were included. 2 patients (8%) had culprit lesions on coronary angiography and underwent percutaneous coronary intervention. 13 patients (52%) demonstrated LGE: (i) *n* = 4 ischemic, (ii) *n* = 4 non-ischemic, and (iii) *n* = 5 ischemic AND non-ischemic. In the 12 LGE-negative patients, mapping revealed diffuse myocardial damage in additional 9 (75%) patients, with a high prevalence of increased T2 values.

**Conclusions:**

Our data show a low prevalence of culprit lesions in troponin-positive stroke patients. However, > 50% of the patients demonstrated myocardial scars (ischemic + non-ischemic) by LGE-CMR. Mapping revealed additional myocardial abnormalities (mostly inflammatory) in the majority of LGE-negative patients. Therefore, a comprehensive CMR protocol gives important insights in the etiology of troponin which might have implications for the further work-up of troponin-positive stroke patients.

## Introduction

Current guidelines recommend the measurement of troponin in the early management of patients with acute ischemic stroke (1). Thereby, increased levels of high-sensitive cardiac troponin I (hs-cTnI) occur in up to 90% of the patients, and are known to be associated with an unfavorable outcome (2). Remarkably, only a minority of patients with elevated troponin values demonstrates culprit lesions on coronary angiography with the need of immediate intervention as the most reasonable cause for troponin release. Therefore, other mechanisms of myocardial injury leading to elevated troponin levels must be present. Myocardial damage of non-ischemic causes, e.g., inflammation, might be an alternative reason. Another explanation may be the concept of stroke-heart syndrome (2), in which cardiac manifestations (e.g., myocardial injury, dysfunction and arrhythmia) are induced by an acute ischemic stroke, potentially mediated by autonomic function and the brain-heart axis (3–5). Beside the presence of an increased troponin level itself, dynamic changes in troponin levels are of paramount importance for the treating physicians, allowing to separate acute myocardial injury (dynamic rise/fall > 20% in serial measurements) from chronic myocardial injury (increased but stable hs-cTnI levels) (6, 7). However, the reason for acute myocardial injury remains often elusive since symptoms, ECG, echocardiography or even coronary angiography might be unspecific or ambiguous (4). Therefore, distinct myocardial tissue characterization to investigate further the reason for myocardial injury would be highly desirable. There is compelling evidence that cardiac magnetic resonance (CMR) is the preferred method for non-invasive myocardial tissue characterization, with its technique of Late Gadolinium Enhancement (LGE) allowing separation between ischemic and non-ischemic myocardial scars. The addition of recent mapping techniques [T1, T2, extracellular volume (ECV)] enables the detection of diffuse, even subtle myocardial processes indicating fibrosis, inflammation, or a combination of both (8–10).

Therefore, we sought to investigate: (1) the prevalence of culprit lesions by coronary angiography, and (2) myocardial tissue abnormalities as a correlate of myocardial injury detected by a comprehensive CMR protocol including LGE and recent mapping techniques in troponin-positive patients with acute ischemic stroke.

## Materials and methods

### Patient population

Thirty-two consecutive patients with acute ischemic stroke and positive troponin were prospectively enrolled if they fulfilled the following criteria: (1) MRI and/or CT proven acute ischemic stroke; (2) repeated measurements of hs-cTnI; (3) no history of coronary artery disease, myocardial infarction, or previous revascularization; (4) coronary angiography (either by x-ray or computed tomography); (5) no contraindications to CMR; and (6) successfully underwent a dedicated CMR protocol including cine, LGE, and recent mapping techniques. Patients fulfilling the latter criteria were enrolled regardless of potential symptoms or ECG abnormalities. Patients with pre-existing comorbidities potentially affecting the myocardium were excluded. Four patients were excluded due to pre-existing cardiomyopathies (*n* = 3 dilative cardiomyopathy and *n* = 1 toxic cardiomyopathy after chemotherapy) and three patients were excluded due to a history of coronary artery disease. Twenty-five complete datasets remained fully evaluable for this study. According to the fourth universal definition of myocardial infarction, patients were categorized by their repeated troponin measurements in: (1) *chronic* myocardial injury (increased but stable troponin levels in terms of rise/fall ≤ 20% in serial measurements), and (2) *acute* myocardial injury (rise/fall of troponin > 20% in serial measurements) (6, 7). Twelve healthy volunteers [5 female (42%), age 51 ± 9 years] served as a control group to define reference values for the CMR mapping sequences (T1 and T2 mapping). The study complies with the Declaration of Helsinki, the local ethics committee (University of Tübingen, Germany) has approved the research protocol and informed consent has been obtained from the subjects.

### Cardiovascular magnetic resonance protocol

Electrocardiography-gated CMR imaging was performed in line with the Society of Cardiovascular Magnetic Resonance/European Cardiovascular Magnetic Resonance recommendations in breath-hold (11) on a 1.5 T Siemens Aera scanner (Siemens Healthineers, Erlangen, Germany). Cine, mapping and LGE short-axis CMR images were acquired every 10 mm (slice thickness 6 mm) from base to apex. Cine CMR was performed using a steady-state free precession sequence. Native T1, T2 mapping and ECV were acquired with product type sequences (MyoMaps, Siemens Healthineers, Erlangen, Germany: T1 MOLLI 5(3)3 sequence; T2 prepared spoiled gradient echo (FLASH), T2 preparations 0, 30, 50 ms), also see Supplementary File. LGE images were acquired on average 10 min after contrast administration with a segmented phase-sensitive Inversion Recovery (PSIR) sequence, inversion time was optimized for dark blood and high contrast in PSIR images (12). The contrast dose was 0.15 mmol/kg gadobutrol (Gadovist, Bayer Vital, Leverkusen, Germany).

### Cardiovascular magnetic resonance analysis

Two experienced investigators analyzed all CMR images together in consensus, blinded to the clinical data. Cine images were evaluated as described previously (11). In brief, endocardial and epicardial borders were outlined on the short-axis cine images. Volumes and ejection fraction (EF) were derived by summation of epicardial and endocardial contours. The LV mass was calculated by subtracting endocardial from epicardial volume at end-diastole and multiplying by 1.05 g/cm^3^. For post-processing and quantification of the LGE and mapping images dedicated software (circle CVI 42, Version 5.11, Calgary, Canada) was used according to Society for Cardiovascular Magnetic Resonance standards (8). Epicardial and endocardial contours were placed manually on all LGE and mapping images which were further analyzed by the 17-segment AHA model (13). LGE was defined of an image intensity level ≥ 2 SD above the mean of remote myocardium; the results were expressed as percentage of myocardial LV mass. LGE was further divided by distribution (linear, focal), and its pattern (epi- and endocardial, midwall) as previously described (8, 9, 14, 15). Ischemic LGE was defined as subendocardial or transmural enhancement; non-ischemic LGE was defined as midwall or subepicardial enhancement.

Abnormal myocardial tissue results were defined as presence of LGE and/or increased T1/T2 values above the 95th percentile of the values of the control group. For ECV, values above ≥ 30% were considered as definitely increased (16–18). Mapping values were determined by two different approaches: (1) entire slice, measuring values in the whole circumference of a midventricular short-axis slice, and (2) segmental, measuring values in 3 short-axis slices (base, midventricular, apical) according to the 17-segment model, allowing the depiction of focal abnormal mapping values in single segments.

### Statistical analysis

Absolute values and percentages were used to describe the patient population. Data distribution was assessed using Shapiro-Wilk test. Variables are presented as mean ± standard deviation (SD) for normally distributed data, medians with interquartile range (IQR) for non-normally distributed data, or absolute numbers (%) for categorical data as appropriate, respectively. Groups with categorical variables were compared by Fisher-Freeman-Halton test. Wilcoxon or *t*-test were used to compare continuous variables. All statistical analyses were performed using JMP (Version 14, SAS Institute Inc., Cary, NC, USA) or SPSS 22.0 (IBM Corp., Armonk, NY, USA).

## Results

### Patient characteristics

Overall, 25 troponin-positive ischemic stroke patients (median age 62 years; 56% male, median hs-cTnI peak 882 ng/L) were enrolled. All patients demonstrated acute myocardial injury defined by a dynamic rise/fall pattern > 20% of troponin levels in serial measurements. Cardiac symptoms were sparse (*n* = 2 chest pain, *n* = 3 dyspnea, *n* = 3 syncope). Most patients (72%) had a history of arterial hypertension. CMR was performed mean 4.4 ± 2.4 days after the acute ischemic stroke. Dividing patients by the presence of LGE, patient characteristics differ only for hs-cTnI and CK levels, with LGE-positive patients demonstrating both higher hs-cTnI and creatine kinase (CK) levels, Table 1. ECG abnormalities were present in 15 patients (60%), comprising ST-segment depression (*n* = 7), negative T waves (*n* = 5), left bundle branch block (*n* = 2), and atrial fibrillation (*n* = 2). No patient demonstrated ST-segment elevation.

**TABLE 1:** Patient characteristics.

	All patients (*n* = 25)	LGE + (*n* = 13)	LGE − (*n* = 12)	*P* value LGE + vs. LGE−
Age, years	62 (15)	62 (13)	61 (17)	0.7
Sex, female	11 (44%)	3 (23%)	8 (67%)	**0.03**
Time to CMR (days)	4.4 ± 2.4	3.8 ± 1.4	5.0 ± 3.1	0.29
***Cardiovascular risk factors***				
Hypertension	18 (72%)	9 (69%)	9 (75%)	0.7
History of smoking	6 (24%)	3 (23%)	3 (25%)	0.9
Hyperlipidemia	10 (40%)	4 (31%)	6 (50%)	0.6
Diabetes	4 (16%)	1 (8%)	3 (25%)	0.2
Family history	2 (8%)	1 (8%)	1 (8%)	0.9
***Ischemic lesion***				
Anterior circulation (ACA + MCA)	19 (76%)	12 (92%)	7 (58%)	0.9
*Insular region right side*	9 (36%)	5 (39%)	4 (33%)	0.9
Posterior circulation	6 (24%)	1 (8%)	5 (42%)	**0.047**
Stroke severity, NIHSS score	2 (7)	3 (8)	1 (5)	0.4
***Stroke etiology***				
TOAST 1	2 (8%)	1 (8%)	1 (8%)	0.9
TOAST 2	7 (28%)	5 (39%)	2 (17%)	0.2
TOAST 3	1 (4%)	0 (0%)	1 (8%)	0.3
TOAST 4	1 (4%)	0 (0%)	1 (8%)	0.3
TOAST 5	14 (56%)	7 (54%)	7 (58%)	0.8
***Biomarkers***				
Baseline hsTn, ng/l	215 (963)	414 (1808)	115 (508)	**0.04**
Maximum hsTn, ng/l	882 (1468)	1280 (1796)	235 (1089)	**0.02**
Time of max hsTn (hrs)	6 (24)	24 (22.5)	3 (24)	0.4
Maximum CK, mg/dl	127 (96)	155 (233)	98 (93)	**0.05**
NT-proBNP, ng/l	1800 (4597)	2029 (4936)	1478 (6595)	0.7
CRP, mg/dl	0.9 (1.4)	0.8 (1.4)	1.0 (1.3)	0.6
***ECG***				
ST-depression	7 (28%)	4 (31%)	3 (25%)	0.7
***Hemodynamics***				
Heart rate	79 (15)	78 (15)	80 (32)	0.5
Systolic BP	156 (38)	159 (41)	154 (54)	0.7
Diastolic BP	85 (23)	90 (23)	84 (24)	0.5
***Renal function***				
GFR (ml/min/1.73 m^2^)	89 ± 35	100 ± 41	77 ± 23	0.1
***Medication at time of CMR***				
Aspirin	17 (68%)	8 (62%)	9 (75%)	0.2
P2Y12-inhibitor	3 (12%)	1 (8%)	2 (17%)	0.5
DOAC	5 (20%)	4 (31%)	1 (8%)	0.5
Statins	21 (76%)	12 (92%)	9 (75%)	0.3
Beta-blockers	14 (56%)	7 (54%)	7 (58%)	0.8
ACE inhibitors	6 (24%)	2 (12%)	4 (33%)	0.3
CCBs	4 (16%)	1 (8%)	3 (25%)	0.3
ARBs	6 (24%)	4 (31%)	2 (17%)	0.5
Diuretics	6 (24%)	2 (15%)	4 (33%)	0.3
Oral antidiabetics	3 (12%)	1 (8%)	2 (17%)	0.5

Values are given as frequency *n* (percentage%), or median (interquartile range). LGE, late gadolinium enhancement; CMR, cardiac magnetic resonance; ACA, anterior cerebral artery, MCA, middle cerebral artery; NIHSS, National Institutes of Health Stroke Scale; TOAST, Trial of Org 10172 in Acute *Stroke* Treatment; hs-cTnI, high sensitivity cardiac troponin I; CK, creatine kinase; NT-proBNP, N-terminal pro-B-type natriuretic peptide; CRP, C-reactive protein; GFR, glomerular filtration rate; ECG, electrocardiogram; BP, blood pressure; GFR, glomerular filtration rate; DOAC, direct oral anticoagulant; ACE, angiotensin converting enzyme; CCBs, calcium channel blockers; ARBs, angiotensin receptor blockers. The statistically significant *p* ≤ 0.05 is represented by bold values.

### Detection of high-grade coronary stenosis (culprit lesion)

Among the 25 patients, only 2 patients (8%) demonstrated high-grade coronary stenosis > 90% (culprit lesion) on coronary angiography with the need of immediate treatment. Both patients reported no cardiac complaints (e.g., angina, dyspnea), but showed ECG abnormalities (negative T waves, and ST-segment depression, respectively) and demonstrated ischemic scars on LGE-CMR correspondent to the area supplied by the high-grade stenotic vessel.

### Functional cardiovascular magnetic resonance parameters and late gadolinium enhancement

**FIGURE 1:**
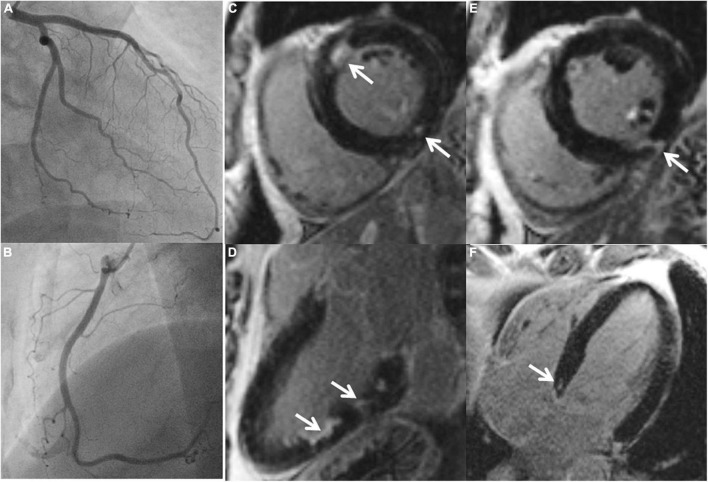
A 45-year-old male with ischemic stroke, unobstructed coronary arteries and multiple LGE lesions. A 45-year old male with ischemic middle cerebral artery (MCA) stroke (NIHSS 8) successfully underwent systemic thrombolysis. Troponin was increased at admission, maximum 1.18 U/l (reference 0.04 U/l), CK maximum 212 ml/l, chest pain was denied. ECG: sinus rhythm, no ST-segment alterations. Echocardiography showed a preserved left ventricular ejection fraction, no wall motion abnormalities. Cardiovascular risk factors: arterial hypertension. Coronary angiography **(A,B)** excluded coronary stenoses. CMR **(C–F)** revealed several areas demonstrating LGE (arrows).

Cardiovascular magnetic resonance revealed an overall preserved left ventricular ejection fraction (mean 57 ± 15%). None of the patients showed apical or midventricular ballooning, suggesting Takotsubo syndrome. 13 patients (52%) demonstrated (mainly focal) LGE with a median total volume of 5% LV mass, Tables 1, 2 and Supplementary Tables A, B. Four patients each showed exclusively nonischemic LGE or ischemic LGE, whereas 5 patients had a combination of non-ischemic and ischemic myocardial scars, also see Figure 1.

**TABLE 2:** CMR findings patients vs. controls.

	All patients (*n* = 25)	Controls	*P* value
***Functional analysis***			
LV-EF (%)	57 ± 15	65 ± 3	**0.01**
LV-EDV (ml)	185 ± 63	161 ± 24	0.1
LV-SV (ml)	99 ± 32	105 ± 18	0.5
***Presence of LGE***	13 (52%)	–	–
LGE total volume (%LV mass)	5% (2–10%)	–	–
LGE + segments	3 (1–7)	–	–
***LGE patterns***			
Ischemic	4 (31%)	–	–
Non-ischemic	4 (31%)	–	–
Combined (ischemic + non-ischemic)	5 (38%)	–	–
***Mapping***			
***Native T1***			
Native T1 mid slice (ms)	1054 ± 44	993 ± 32	**0.0003**
Native T1 entire slice positive (patient frequency, %)	15 (60%)	–	–
Native T1 ≥ 1 positive segment (patient frequency, %)	22 (88%)	–	–
Native T1 positive segments (median, IQR)	4 (3–6)	–	–
***ECV***			
ECV (median, IQR)	30% (28–33%)	–	–
ECV entire slice positive (patient frequency, %)	9 (36%)	–	–
ECV ≥ 1 positive segment (patient frequency, %)	17 (68%)	–	–
ECV positive segments (median, IQR)	2 (1–5)	–	–
***T2***			
T2 mid slice (ms)	52 ± 4	47 ± 2	**0.004**
T2 entire slice positive (patient frequency, %)	14 (56%)	–	–
T2 ≥ 1 positive segment (patient frequency, %)	19 (76%)	–	–
T2 positive segments (median, IQR)	3.5 (1–6)	–	–

Values are given as frequency *n* (percentage%), mean (SD = standard deviation); or median (IQR = interquartile range). LV, left ventricle; EF, ejection fraction; EDV, end-diastolic volume; SV, stroke volume; LGE, late gadolinium enhancement; ECV, extracellular volume. The statistically significant *p* ≤ 0.05 is represented by bold values.

### Cardiovascular magnetic resonance mapping techniques

Troponin-positive stroke patients demonstrated increased native T1 (1,054 vs. 993 ms) and T2 mapping values (52 vs. 47 ms) compared to controls, *p* < 0.001, and *p* = 0.004, respectively, Table 2. By the entire slice approach, 60% (*n* = 15) of the patients had a native T1 above the 95th percentile of the healthy control group, fulfilling the criteria of a definite abnormal T1 mapping value. For T2 mapping, 56% (*n* = 14) of the patients demonstrated a T2 value above the 95th percentile of the healthy control group. For ECV, 36% (*n* = 9) patients demonstrated a value ≥ 30% indicating abnormal increased extracellular volume.

### Mapping in late gadolinium enhancement-negative patients

**FIGURE 2:**
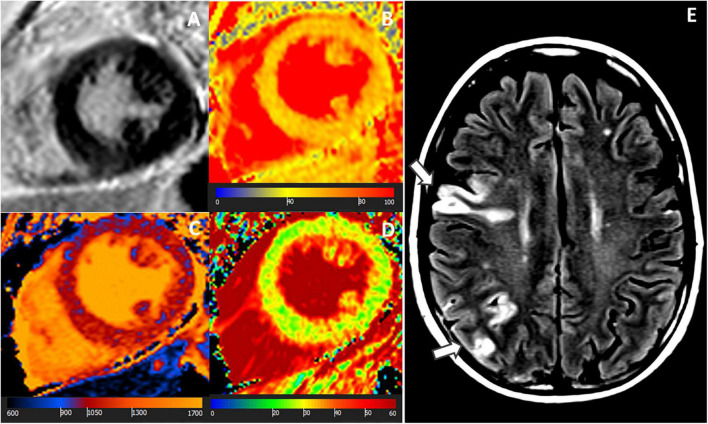
A 58-year-old female with ischemic stroke, negative LGE but abnormal mapping values. CMR images: **(A)** LGE-negative, **(B)** increased T1 (1,064 ms), **(C)** increased T2 (56 ms), **(D)** increased ECV (30%) in an identical midventricular short-axis slice. **(E)** Cerebral MR showed multiple acute ischemic cortical and sub-cortical lesions in right middle and posterior cerebral artery territories (arrows). Coronary angiography revealed no coronary stenosis.

**FIGURE 3:**
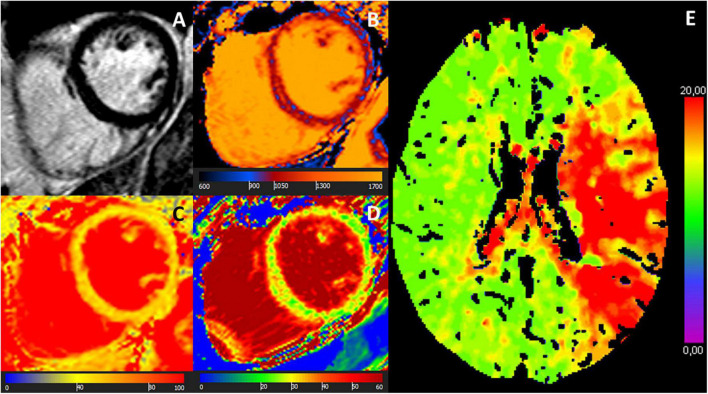
MR images of a 68-year-old female with troponin-positive ischemic stroke and unobstructed coronary arteries. **(A)** LGE-negative, **(B)** increased T1 (1,074 ms), **(C)** increased T2 (52 ms), **(D)** increased ECV (30%) in an identical midventricular short-axis slice. **(E)** Cerebral volume perfusion CT at admission showed prolonged time-to-peak perfusion in the territory of the acute occluded left middle cerebral artery (red area).

12 (48%) of the patients were LGE-negative. Out of these, 75% (*n* = 9) demonstrated at least one abnormal mapping value (native T1, T2, ECV), Table 3. By the entire slice approach, 7 patients demonstrated increased native T1 values, 7 patients showed increased T2 values, and 4 patients had an increased ECV. By the 17-segment approach, 9 patients demonstrated increased native T1 values, 7 patients showed increased T2 values, and 8 patients had an increased ECV, Table 3 and Figures 2, 3. The majority of patients with increased native T1 values demonstrated also increased T2 (and ECV) values. Compared to native T1 and ECV, the segmental approach for T2 analysis did not identify additional patients with increased values.

**TABLE 3:** CMR findings in LGE negative patients: Additional value of mapping.

LGE-negative patients (*n* = 12)	Entire T1	Segmental T1	Entire ECV	Segmental ECV	Entire T2	Segmental T2
1	+	+	+	+	+	+
2	−	−	−	−	−	−
3	−	−	−	−	−	−
4	−	−	−	−	−	−
5	+	+	+	+	+	+
6	−	+	−	+	−	−
7	+	+	+	+	+	+
8	+	+	−	+	+	+
9	+	+	−	−	−	−
10	−	+	−	+	+	+
11	+	+	+	+	+	+
12	+	+	−	+	+	+
**n (% patients)**	7 (58%)	9 (75%)	4 (33%)	8 (67%)	7 (58%)	7 (58%)

+ = positive finding; entire (slice) or at least one segment (segmental) value above 95th percentile of control group. − = negative finding.

Three out of the 25 troponin-positive stroke patients (12%) had no myocardial tissue abnormality revealed by a comprehensive CMR approach including LGE and mapping techniques.

## Discussion

This is the first prospective multi-component CMR study to characterize non-invasively myocardium in troponin-positive ischemic stroke patients. As expected, the number of troponin-positive patients who need immediate treatment of a high-grade coronary stenosis by percutaneous coronary intervention was low (8%). Conversely, we found a high rate of myocardial tissue abnormalities (in 88% of the patients) with a preponderance for non-ischemic myocardial lesions, in particular inflammation, as diagnosed by a multi-component CMR protocol (LGE, T1 and T2 mapping, ECV). Our findings suggest substantial diagnostic value for a multi-component CMR in the challenging work-up of patients with troponin-positive ischemic stroke beside the careful evaluation of patient symptoms, ECG, echo and coronary angiography, respectively.

### Detection of high-grade coronary stenosis (culprit lesion)

Raised troponin levels, which are associated with an unfavorable outcome, are detected in up to 90% of stroke patients (2). Acute “dynamic” elevation of troponin (characterized by the rise or fall in repeated measurements) is present in 30–60% of the patients. Despite several theories, the exact mechanisms of myocardial injury resulting in increased troponin levels are still unknown and to date, no specific diagnostic or therapeutic algorithms are established beside general cardiology guidelines for the work-up of troponin-positive ischemic stroke patients (2, 4, 6, 7). Significant coronary artery disease seems not to be main driver of increased troponin levels in the majority of patients (19), lowering the role of invasive coronary angiography as a “*sine qua non*” diagnostic tool, and underlining the unmet need of further evaluation in these high-risk patients.

Among the 25 patients, only 2 patients (8%) demonstrated high-grade stenosis > 90% (culprit lesion) on coronary angiography. Choosing wisely those stroke patients who might benefit from invasive coronary angiography is further limited by the potential absence of symptoms which might be masked by the acute neurological disorder. In this study, both patients with high-grade coronary stenosis reported no cardiac complaints, but showed ECG abnormalities and demonstrated ischemic scars on LGE-CMR, corresponding to the area supplied by the stenotic vessel. Therefore, those two patients would have been classified as having CAD on the basis of CMR alone, underscoring the value of non-invasive CMR for detection of both ischemic and non-ischemic cardiomyopathies.

Patients with non-ST-segment elevation myocardial infarction often routinely undergo invasive coronary angiography, and also in the general population many of these patients do not have obstructive coronary artery disease with the need of immediate invasive treatment. Recent data suggest that vasodilator stress CMR or cardiac computed tomography first in the diagnostic process of these patients might be a safe gatekeeper for invasive coronary angiography (20). The increasing role of (stress) CMR and/or cardiac computed tomography as non-invasive diagnostic imaging tools in the evaluation of patients with non-ST-segment elevation myocardial infarction is also acknowledged by current guidelines (21). Beside the detection of myocardial ischemia to detect functional coronary stenosis by stress CMR, distinct myocardial tissue characterization provided by CMR techniques (LGE and mapping), allow reliable identification of other etiologies causing myocardial injury (e.g., non-ischemic such as acute myocarditis, Takotsubo Syndrome, and other conditions) in the majority of cases (22).

### Myocardial tissue characterization by comprehensive cardiovascular magnetic resonance

Apart from acute thrombotic CAD, a mismatch of oxygen supply and demand is considered as a potential reason for increased troponin levels. Various other factors such as rhythm disturbances, blood pressure instabilities, respiratory failure and renal failure may also contribute to a rise in troponin levels (6). In our study, 13 out of 25 patients (52%) demonstrated (mainly focal) myocardial scars (LGE) with an overall low total volume of 5% LV mass, Tables 1, 2 and Supplementary Tables A, B. Out of these, 4 patients each showed exclusively non-ischemic LGE or ischemic LGE, respectively, whereas 5 patients had a combination of non-ischemic and ischemic myocardial lesions, suggesting a substantial rate of myocardial injury not caused by thrombotic coronary lesions. Dividing patients by the presence of LGE revealed that LGE-positive patients demonstrated both higher hs-cTnI and creatine kinase (CK) levels, indicating a more severe and irreversible myocardial damage in the LGE-positives.

Suggested mechanisms of myocardial injury in stroke patients are excessive sympathetic response by the central autonomic nervous system and metabolic and oxidative stress pathways which might induce necrosis and inflammatory reaction. Noradrenaline seems to be a main driver of myocardial injury, damages cardiomyocytes, stimulates macrophages, and leads to activation of fibroblasts to myofibroblasts (4, 6, 23). Furthermore, neuronal death may result in local cerebral inflammation by activation of microglia and the production of cytokines and chemokines (4). Another potential mechanism is impaired mitochondrial function induced by the acute cerebrovascular event (4).

Compared to LGE alone, which indicates irreversible myocardial scar, CMR mapping techniques delineate better between fibrotic and inflammatory myocardial alterations; native T1 is unspecific for fibrosis or inflammation, but T2 is sensitive to myocardial edema indicating myocardial inflammation, allowing a better distinction of chronic from acute myocardial processes. In our cohort, we found both increased native T1 (1,054 vs. 993 ms) and T2 mapping values (52 vs. 47 ms) in troponin-positive stroke patients vs. controls, supporting the role of co-existing necrosis and inflammation, Table 2. Specifically, native T1 values were definitely increased (above the 95th percentile of the values of the control group) in 60% (*n* = 15), and T2 values were increased in 56% (*n* = 14) of the patients. The high prevalence of myocardial edema suggests rather an acute myocardial process (associated with the acute event of ischemic stroke) than prior existing myocardial abnormalities. Additionally, we tried to minimize the potential of prior (established) myocardial lesions by excluding patients with a history of CAD, myocardial infarction, or previous revascularization.

### Diffuse myocardial alterations in late gadolinium enhancement-negative patients

12 (48%) of the patients demonstrated no focal fibrosis (LGE-negative), drawing attention to the CMR mapping techniques, which allow the detection of diffuse, potentially still reversible myocardial alterations. Indeed, 75% (*n* = 9) of the patients demonstrated abnormal mapping values, suggesting diffuse myocardial processes (such as fibrosis and/or inflammation) which might be triggered, e.g., by sympathetic overactivation and release of interleukin 1 (IL-1) (4, 5). Measuring mapping values in an entire midventricular slice, 7 patients demonstrated increased native T1 values (representing diffuse fibrosis or inflammation), and 7 patients showed increased T2 values (suggesting diffuse myocardial inflammation). In 6 of these patients (86%), T1 and T2 were both increased, suggesting substantial diffuse, potential reversible, inflammatory myocardial processes even in patients without irreversible scar (LGE negative) as an explanation for increased troponin levels, Table 3.

Focusing on focal myocardial abnormalities according to the established segment AHA model, 9 patients demonstrated increased native T1 values. Out of these, 7 patients showed increased T2 values, and 8 patients had an increased ECV, Table 3. Thus, an increased native T1 value in at least one segment seems to be the most sensitive parameter for the detection of myocardial abnormality. Again, concomitant increased T2 values as sign of myocardial edema underline myocardial inflammation as an important cornerstone in the pathophysiology of troponin-positive stroke patients.

Hence, extending the CMR protocol (beside functional analysis and LGE) by mapping techniques seem to be a promising way to detect even subtle myocardial alterations in a high percentage of troponin-positive ischemic stroke patients, illustrating non-invasively the pathophysiologic processes of myocardial fibrosis and inflammation which seem to be present in a substantial part of these patients. Overall, only 3 of the 25 troponin-positive stroke patients (12%) had no myocardial tissue abnormality investigated by a comprehensive CMR approach and in these candidates increased troponin might be explained by some other reasons than myocardial injury. Troponin elevations might be explained in some patients by high blood pressure or heart rate (extrasystoles, paroxysmal atrial fibrillation) or stress due to stroke, factors which can also mitigate myocardial injury. This could also be a mechanism in patients with normal mapping (T2) and without edema.

Our study underlines the potential of a multi-component CMR protocol as a dedicated non-invasive myocardial tissue imaging tool in the work-up of troponin-positive patients beside coronary angiography, which revealed only a low prevalence of coronary stenosis attributable for increased troponin levels. One explanation for the high number of myocardial abnormalities detected by CMR might be the fact that all patients demonstrated acute myocardial injury, as defined by rise/fall of troponin > 20% in serial measurements. One might argue that in patients with increased, but stable, troponin levels (= chronic myocardial injury), other reasons than direct myocardial injury (displayed by CMR) might be more frequent. However, patients with ischemic stroke and acute myocardial injury, as presented by this study, are the most challenging candidates for both cardiologist and neurologist in the everyday clinical routine, underlining the need of close interaction between both disciplines, taking into account the individual patient clinics and history, laboratory parameters, ECG, and echocardiography. However, due to various reasons of troponin liberation, increased troponin levels in some patients might not even be explained despite knowledge of the patient clinics, laboratory markers, ECG, coronary angiography and even CMR. Important to mention that CMR should not be regarded as a substitute for coronary angiography in stroke patients with acute coronary syndrome as defined by current acute coronary syndrome guidelines, but might rather represent a valuable tool in cases where increased troponin levels are elusive and patient history, ECG and echocardiography point toward an overall low risk of CAD.

According to other forms of cardiomyopathies, CMR mapping techniques including native T1, T2, and ECV might be promising not only for diagnosis but also for risk prediction since abnormalities (reflected by increased values for native T1, T2 or ECV) seem to be associated with a higher rate of major adverse cardiovascular events (24, 25). Therefore, results of a comprehensive CMR approach, as performed in this study, might also have implications for the need of a closer cardiovascular monitoring regimen in patients with troponin-positive acute ischemic stroke, which has to be investigated by other studies.

### Limitations

Patients in this study were middle-aged (median age 62 years) and therefore not comparable to general ischemic stroke studies where patients are older. However, inclusion of patients at a relatively younger age lowers the risk to detect prior existing myocardial abnormalities (e.g., previous myocardial infarction), which are not associated with the acute cerebrovascular event. However, we cannot fully exclude that some patients might demonstrate myocardial tissue abnormalities, which might not be directly linked to the acute ischemic cerebrovascular event, although the high rate of myocardial inflammation reported by this study rather suggest acute myocardial processes. No specific inflammatory proteins were measured by this study.

Moreover, the population in this single-center study is relatively small. However, to date this study represents the first study in troponin-positive ischemic stroke patients who underwent both coronary angiography and a multi-component CMR including LGE and mapping techniques.

Another limitation is the need of intermittent breath-holding during the CMR scan. This might be hindered in patients with severe neurological disorders (large infarctions, aphasia) yielding in impaired imaging quality.

Findings were investigated in an ischemic stroke population with acute myocardial injury, so the results of this study cannot be transferred to ischemic stroke populations with chronic myocardial injury. One might argue that in the latter group the prevalence of myocardial abnormalities, detected by comprehensive CMR, might be lower. However, acute myocardial injury is associated with a more severe adverse outcome. Therefore, detecting the underlying pathology is of utmost importance since it may affect patient outcome (4, 26).

## Conclusion

Increased troponin levels are a common finding in ischemic stroke patients. However, the prevalence of significant coronary stenosis with the need of immediate treatment (culprit lesion) as an explanation for increased troponin levels is low. A multi-component CMR protocol, including LGE and recent CMR mapping techniques, provides an optimal non-invasive imaging tool to (1) detect and separate ischemic scars from non-ischemic scars, and (2) detect subtle processes of myocardial fibrosis and/or inflammation which seem to be more prevalent in ischemic stroke patients than acute thrombotic coronary lesions. Our results may have implications for the challenging clinical work-up in troponin-positive patients with acute ischemic stroke, illustrating both ischemic or non-ischemic myocardial damage non-invasively. However, despite excellent non-invasive myocardial tissue characterization by CMR and an overall low prevalence of culprit lesions in troponin-positive stroke patients, the treating physician should be aware of patient symptoms, ECG, echocardiography and the patient probability for CAD acknowledging current guidelines. Coronary angiography is still an indispensable tool but needs to be performed in the minority of troponin-positive patients with acute ischemic stroke.

## Data availability statement

The raw data supporting the conclusions of this article will be made available by the authors, without undue reservation.

## Ethics statement

The studies involving human participants were reviewed and approved by the University of Tübingen, Germany. The patients/participants provided their written informed consent to participate in this study.

## Author contributions

AM, LN, KM, LM, TG, JK, PK, and SG contributed to the idea and design of the report, acquired and analyzed the data, and wrote the manuscript. SP, MK, KF, UE, and FH contributed to the design of the study, analysis of the data, and revision of the manuscript. HM, UZ, KN, and MG revised the manuscript. All authors read and approved the final manuscript.

## Abbreviations

ACA: anterior cerebral artery
AHA: American Heart Association
CAD: coronary artery disease
CMR: cardiovascular magnetic resonance
CK: creatine kinase
CRP: C-reactive protein
ECG: electrocardiogram
ECV: extracellular volume
EDV: end-diastolic volume
EF: ejection fraction
hs-cTnI: high sensitivity cardiac troponin I
IQR: interquartile range
LGE: late gadolinium enhancement
LV: left ventricle
MCA: middle cerebral artery
NIHSS: National Institute of Health Stroke Scale
TOAST: Trial of Org 10172 in Acute Stroke Treatment
SD: standard deviation
SV: stroke volume.
