# Supplementary material for: TRoponin of Unknown origin in STroke evaluated by multi-component cardiac Magnetic resonance Imaging – The TRUST-MI study
Source: Front Cardiovasc Med. 2022 Sep 30;9:989376. doi: 10.3389/fcvm.2022.989376 (PMC9561415; doi:10.3389/fcvm.2022.989376)
Supplement: Supplementary file 1 [file Data_Sheet_1.DOCX]

**Supplementary table A:**

**LGE by 17-segment AHA model in n=13 LGE-positive patients**

| - segment 1 - segment 2 - segment 3 - segment 4 - segment 5 - segment 6 - segment 7 - segment 8 - segment 9 - segment 10 - segment 11 - segment 12 - segment 13 - segment 14 - segment 15 - segment 16 - segment 17 | 2 (15%)  5 (38%)  5 (38%)  2 (15%)  4 (31%)  2 (15%)  2 (15%)  6 (46%)  2 (15%)  3 (23%)  4 (31%)  2 (15%)  1 (8%)  -  6 (46%)  3 (23%)  - |
| --- | --- |

Values are given as patient frequency (%)

**Supplementary table B:**

**LGE patterns in n=13 LGE-positive patients**

| ***LGE patterns (multiple possible)*** |  |
| --- | --- |
| subendocardial | 4 (31%) |
| transmural | 4 (31%) |
| subepicardial | 5 (38%) |
| midwall | 6 (46%) |
| focal | 10 (77%) |
| linear | 2 (15%) |
