# Supplementary material for: TRoponin of Unknown origin in STroke evaluated by multi-component cardiac Magnetic resonance Imaging – The TRUST-MI study
Source: Front Cardiovasc Med. 2022 Sep 30;9:989376. doi: 10.3389/fcvm.2022.989376 (PMC9561415; doi:10.3389/fcvm.2022.989376)
Supplement: Supplementary file 2 [file Data_Sheet_2.DOCX]

**Supplementary Material**

**CMR Mapping Acquisition Parameters**

**T1 mapping:**

**Native T1 mapping was acquired with a product type sequence in breath-hold (MyoMaps, Siemens Healthineers, Erlangen, Germany: T1 MOLLI sequence with 5(3)3 acquisition scheme. Slice thickness was 8 mm, spacing between slices 10 mm (2 mm gap), acquisition base to apex covering the entire left ventricle. Matrix 256 x 142; Field of View 284 x 340; repetition time (TR) depending on the cardiac cycle; echo time (TE) 1.15 ms.**

**T2 mapping:**

**T2 mapping was acquired with a product type sequence in breath-hold (MyoMaps, Siemens Healthineers, Erlangen, Germany: T2 prepared spoiled gradient echo (FLASH), with different T2 preparations at 0 ms, 30 ms, 50 ms. Slice thickness was 8 mm, spacing between slices 10 mm (2 mm gap), acquisition base to apex covering the entire left ventricle. Matrix 256 x 142; Field of View 284 x 340; repetition time (TR) depending on the cardiac cycle; echo time (TE) 1.32 ms. All short-axis slices were acquired in identical slice positions.**
